# Factors Associated With the Severity of Sexual Abuse Among Adolescents in Reception Centers in Kinshasa, Democratic Republic of the Congo: A Cross-Sectional Study

**DOI:** 10.7759/cureus.109742

**Published:** 2026-05-27

**Authors:** Eric Muteba Vukulu, Thierry Matonda-ma-Nzuzi, Adelin N'situ Mankubu, Pascal Tshibuabua Mutshipayi, Alfred Sodi Magudigana, Nathan Tsengele, Damien Junior Mayemba Nsundi, Said Mbuku Nguala, Ally Ndjukendi Omba

**Affiliations:** 1 Department of Psychiatry, Neuropsychopathology Center, Faculty of Medicine, University of Kinshasa, Kinshasa, COD; 2 Department of Neurology, Neuropsychopathology Center, Faculty of Medicine, University of Kinshasa, Kinshasa, COD

**Keywords:** adolescents, associated factors, democratic republic of the congo, kinshasa, reception centers, sexual abuse

## Abstract

Introduction

Sexual abuse among adolescents is associated with significant psychosocial and mental health consequences. In the Democratic Republic of the Congo (DRC), data on factors associated with the severity of these abuses among vulnerable adolescents remain limited. This study aimed to identify factors associated with the severity of sexual abuse among adolescents attending reception centers in Kinshasa.

Methods

A cross-sectional study was conducted from April to August 2025 among 195 adolescents aged 12-18 years recruited from two reception centers in Kinshasa. Sexual abuse was assessed using the corresponding subscale of the Childhood Trauma Questionnaire-Short Form (CTQ-SF), psychological distress using the General Health Questionnaire-28 (GHQ-28), and self-esteem using the Rosenberg Self-Esteem Scale (RSES). Associations were examined using appropriate bivariate tests, followed by ordinal logistic regression. Statistical significance was set at p < 0.05.

Results

Overall, 56.9% of the adolescents reported experiencing sexual abuse, including 12.3% in mild form, 26.2% moderate, and 18.5% severe. In bivariate analysis, the severity of sexual abuse was associated with age, type of center, family structure, psychological distress, self-esteem, and cannabis and tobacco use. After adjustment, the factors independently associated with higher severity were female sex (adjusted odds ratio (aOR) = 1.78; 95% confidence interval (CI): 1.00-3.16), attachment to the mother (aOR = 5.12; 95% CI: 1.74-15.07), attachment to a third person (aOR = 3.34; 95% CI: 1.06-10.49), reconstituted, extended, and nuclear family structures, and cannabis use (aOR = 3.49; 95% CI: 1.56-7.81). An inverse statistical association was observed between alcohol consumption and severity of sexual abuse after adjustment.

Conclusions

Sexual abuse is common among adolescents attending reception centers in Kinshasa. Its severity is associated with familial, relational, and behavioral factors, highlighting the need for multisectoral interventions for prevention, screening, and psychosocial care adapted to this vulnerable population. However, all findings should be interpreted with substantial caution because the assessment instruments used in this study have not undergone formal cross-cultural validation or psychometric adaptation in the Democratic Republic of the Congo.

## Introduction

Sexual abuse refers to the involvement of a child or adolescent in sexual activity that he or she does not fully comprehend, is unable to give informed consent to, or for which the child is not developmentally prepared, and that violates the laws or social taboos of society [1]. Children are generally defined as individuals under 18 years of age, while adolescence refers to the age range of 10-19 years according to the World Health Organization [2]. The present study focused on adolescents aged 12-18 years. The severity of sexual abuse was defined according to the frequency and intensity of experiences measured by the sexual abuse subscale of the Childhood Trauma Questionnaire-Short Form (CTQ-SF) [3].

Sexual abuse among children and adolescents constitutes a major public health problem worldwide due to its high prevalence and lasting consequences on development. According to meta-analyses, between 8% and 31% of girls and between 3% and 17% of boys are victims of sexual abuse before the age of 18 [4]. These experiences are associated with significant consequences on psychosocial, behavioral, and health levels, as well as an increased risk of mental disorders in the short and long term [4-7]. Victims frequently present with psychological distress, including anxiety, depression, and impaired self-esteem [6,8]. They are also more often exposed to risk behaviors, particularly the use of psychoactive substances [9-13].

Sexual abuse is influenced by multiple factors, including individual, familial, and environmental determinants. Sex and age are associated with vulnerability, with girls being more exposed to severe forms [4]. Furthermore, certain family configurations, particularly situations of instability or reconstituted families, are associated with an increased risk of exposure [14-18]. Insecure attachment patterns are associated with increased vulnerability and limited adaptive capacities in the face of traumatic experiences [18].

Environmental factors play a central role, particularly in contexts of precariousness. Adolescents in street situations or attending reception centers are exposed to a lack of family protection, unstable living conditions, and high social vulnerability, which are associated with the occurrence and severity of abuse [3,19]. The absence of adult supervision, economic dependence, exposure to community or institutional violence, early use of psychoactive substances, and limited access to protection and health services are also associated with increased vulnerability [20-24].

In the Democratic Republic of the Congo (DRC), sexual abuse of minors is a worrying and still insufficiently documented phenomenon. Thousands of cases of sexual violence against children were reported in 2025 [24]. Although particularly frequent in conflict zones, these forms of violence also affect urban environments such as Kinshasa, where precariousness and family instability reinforce vulnerability [13]. Local studies report a high prevalence of lifetime sexual violence among adolescents [25,26].

Despite these observations, data remain limited regarding factors associated with the severity of sexual abuse, particularly among adolescents in situations of urban vulnerability. Thus, the present study aims to determine the factors associated with the severity of sexual abuse among adolescents attending reception centers in Kinshasa.

## Materials and methods

Study type, period, and setting

This is a cross-sectional study conducted from April to August 2025 in Kinshasa. This study employed a cross-sectional design to allow the assessment of the prevalence of sexual abuse and to explore its associated factors among adolescents attending reception centers. However, due to the cross-sectional design, causal relationships cannot be established because exposure and outcome were measured simultaneously.

The study was carried out in two reception centers of the Association Sans But Lucratif (ASBL) Œuvre de Suivi, Éducation et Protection des Enfants de la Rue (OSEPER): the open center Sainte Famille, located in the Mpudi neighborhood (Matete commune), and the closed center Esengo, located on By-pass Avenue (Lemba commune). OSEPER is an organization working in the prevention, psychosocial support, vocational training, and family reintegration of street children.

Study population

The study population consisted of adolescents aged 12-18 years attending the two selected reception centers during the study period. This age range corresponded to the population hosted in these structures.

**Figure 1:**
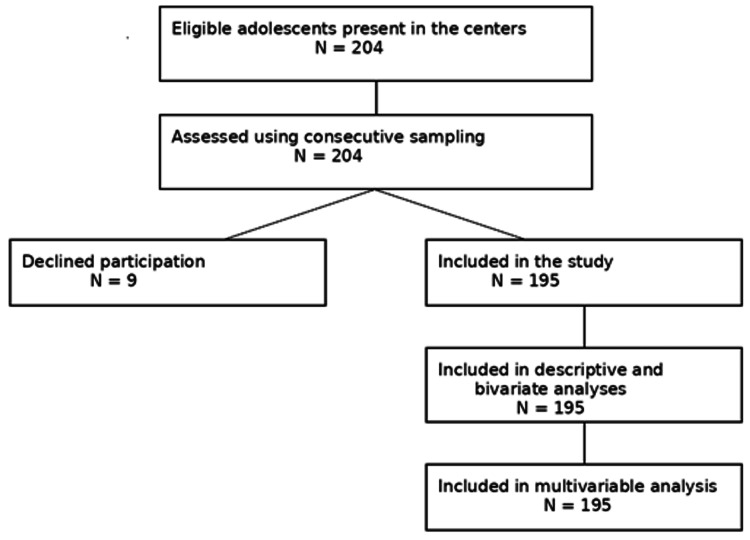
Participant flow diagram

A total of 204 eligible adolescents present in the centers at the time of data collection were assessed using consecutive sampling. Of these, 195 adolescents were included in the study, while 9 refused to participate (Figure 1). The main reason for refusal was the belief that their personal information would be shared with international organizations. Adolescents in a state of intoxication with a psychoactive substance at the time of the interview were re-evaluated later, outside of any consumption.

Consecutive sampling was applied by approaching all eligible adolescents who were present in the two centers during the data collection period until the final sample was obtained. Data were collected through face-to-face interviews conducted in a private setting within each center to ensure confidentiality. The questionnaires were administered in French by trained investigators familiar with adolescent mental health research and ethical procedures for sensitive topics. Before data collection, interviewers were briefed on confidentiality, non-judgmental communication, and referral procedures in case of emotional distress.

Study variables

The dependent variable was the severity of sexual abuse assessed using the Childhood Trauma Questionnaire-Short Form (CTQ-SF) subscale.

Independent variables included sociodemographic, psychological, and behavioral variables. Sociodemographic variables included age, sex, religion, schooling, type of family structure, place of residence before joining the center, type of center attended (open/closed), and length of stay in the center. Psychological variables included the attachment figure, psychological distress, and self-esteem. The behavioral variable included the use of psychoactive substances (alcohol, tobacco, and cannabis).

Operational definition

The type of reception center was categorized as either open or closed. An open center refers to a facility where adolescents attend during the day and are free to enter and leave, with limited supervision and continued exposure to the street environment. A closed center refers to a residential facility where adolescents are accommodated full-time under continuous supervision, with restricted mobility outside the center and structured daily routines, including educational and psychosocial activities.

Measurement instruments

Childhood Trauma Questionnaire-Short Form (CTQ-SF)

Childhood Trauma Questionnaire-Short Form (CTQ-SF) [3,27] is a self-report instrument that identifies different types of maltreatment through five subscales: emotional abuse, physical abuse, sexual abuse, emotional neglect, and physical neglect, with 28 items. Sexual abuse was assessed using only the sexual abuse subscale of the CTQ-SF; the other subscales were not used in this study. This subscale consists of five items rated on a 5-point Likert scale (1 = never true to 5 = very often true), yielding a total score ranging from 5 to 25.

The severity of sexual abuse was categorized according to Bernstein and Fink (1998) [3] as follows: none to minimal = 5, low to moderate = 6-7, moderate to severe = 8-12, and severe to extreme ≥13.

The CTQ has good psychometric properties, with test-retest reliability ranging from 0.79 to 0.86 and internal consistency varying from 0.66 to 0.92. In the present sample, this subscale demonstrated good internal consistency (Cronbach’s α = 0.859).

General Health Questionnaire-28 (GHQ-28)

Psychological distress was assessed using the 28-item General Health Questionnaire (GHQ-28) [28], which explores four dimensions: somatic complaints, anxiety/insomnia, social dysfunction, and depressive symptoms.

The global score is obtained by summing the responses, using a dichotomous scoring (0-0-1-1). A cutoff of 4/5 was used to define psychological distress.

In the present sample, the scale demonstrated excellent internal consistency (Cronbach’s α = 0.927).

Rosenberg Self-Esteem Scale (RSES)

Self-esteem was assessed using the Rosenberg Self-Esteem Scale [29], consisting of 10 items rated on a 4-point Likert scale. The total score ranges from 10 to 40, with higher scores indicating better self-esteem.

This scale has good reliability (Cronbach’s α between 0.77 and 0.88) and satisfactory test-retest stability. In the present sample, this scale showed acceptable internal consistency (Cronbach’s α = 0.802).

The instruments were used in accordance with copyright regulations. No questionnaire items are reproduced in this manuscript.

Attachment Figure

The attachment figure was assessed using a self-reported item asking participants to identify the person to whom they feel most emotionally attached. The response options were predefined and included father, mother, both parents, or a third person (e.g., a relative, guardian, or another significant adult).

Statistical processing and analysis

Data were entered and analyzed using IBM SPSS Statistics version 20.0 (IBM Corp., Armonk, NY). Quantitative variables were described by their means and standard deviations (SD) (or medians and interquartile ranges in case of non-normal distribution), and qualitative variables by their frequencies and percentages.

Bivariate Analysis

Associations between the severity of sexual abuse and explanatory variables were examined using Pearson’s Chi-square test or Fisher’s exact test. For tables larger than 2×2, a significant omnibus test led to post hoc analysis based on adjusted residuals with Bonferroni correction. A priori Helmert orthogonal contrast tests were used to compare mean differences between groups according to severity level.

Multivariate Analysis

An ordinal logistic regression was performed to identify factors associated with the severity of sexual abuse. Variables introduced into the model were those that were clinically relevant and those associated in bivariate analysis with a threshold of p < 0.20.

No internal missing data were observed for the variables included in the analyses.

Results were presented as adjusted odds ratios (aOR) with their 95% confidence intervals (CI). The proportional odds assumption was tested to verify the adequacy of the model.

Given the cross-sectional nature of the study and the temporal ambiguity between certain psychological variables (psychological distress and self-esteem) and the severity of sexual abuse, these variables were not included in the multivariate model to avoid overadjustment bias related to variables potentially downstream of the outcome.

Ethical considerations

The study received approval from the Ethics Committee of the School of Public Health of the University of Kinshasa (approval number: ESP/CE/93/2025). Participation was voluntary and based on informed consent from the adolescents, after authorization from the center supervisors. Participants were informed of the study objectives and their right to withdraw at any time without consequence. Data collection was conducted with respect for the confidentiality, dignity, and privacy of the participants.

## Results

Prevalence of sexual abuse

**Figure 2:**
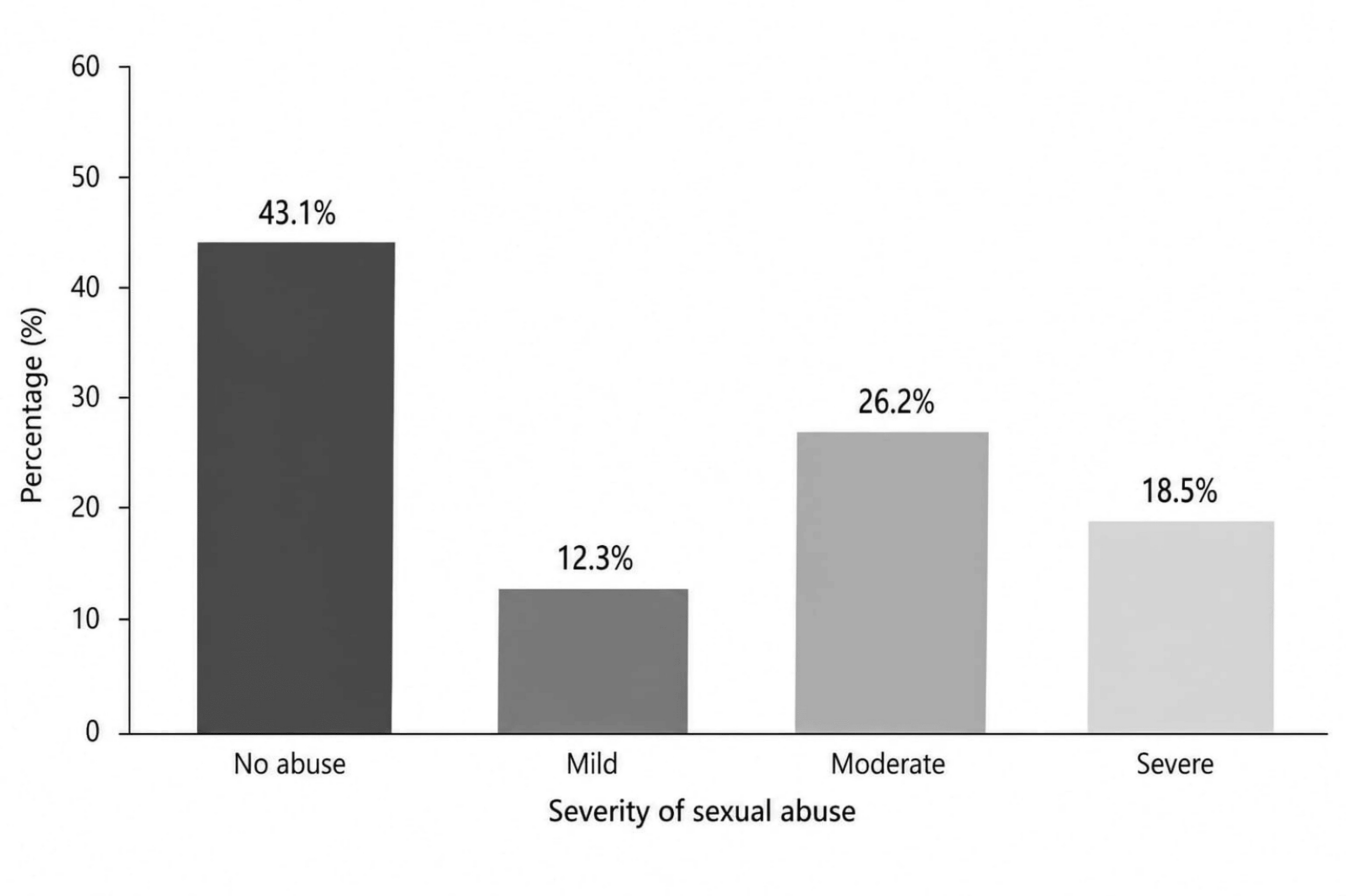
Distribution of adolescents by severity of sexual abuse (N = 195)

Among the 195 adolescents included, 111 (56.9%) reported having experienced at least one form of sexual abuse. The distribution according to severity showed that 24 (12.3%) had mild abuse, 51 (26.2%) moderate, and 36 (18.5%) severe, while 84 (43.1%) reported no abuse (Figure 2).

Sociodemographic characteristics and severity of sexual abuse

The mean age of the participants was 15.05 ± 1.52 years, with a male predominance of 113 (57.9%). The majority were Catholic (161, 82.6%), and 135 (69.2%) were in school. Approximately one-quarter of the adolescents came from reconstituted families, and 120 (61.5%) attended a closed center.

Orthogonal contrast analyses showed that adolescents who were victims of sexual abuse had a significantly higher mean age than those who had not experienced it (t = 2.50; df = 168.68; p = 0.014).

A significant association was observed between the type of institution and the severity of sexual abuse (χ² = 9.99; df = 3; p = 0.019). Post hoc analyses, based on adjusted residuals with Bonferroni correction (α corrected = 0.00625), showed a significantly higher proportion of absence of severe abuse among adolescents attending closed centers (p = 0.0022).

Family structure was also significantly associated with the severity of sexual abuse (χ² = 21.13; df = 9; p = 0.012). Analyses of adjusted residuals (α corrected = 0.0031) highlighted a significantly higher frequency of severe abuse among adolescents from reconstituted and extended families (p = 0.0005).

Finally, the length of stay in the centers differed significantly according to the severity of abuse (t = 2.20; df = 53.27; p = 0.032), with longer durations among adolescents who had experienced severe abuse (Table 1).

**Table 1:** Association between sociodemographic characteristics and sexual abuse SD: standard deviation

Variables	All (number (%))	Severity of sexual abuse				P
		None	Low	Moderate	Severe	
Age mean ± SD (years)	15.1 ± 1.5	14.8 ± 1.4	15.5 ± 1.2	15.16 ± 1.52	15.33 ± 1.69	Significant
Sex						0.185
Male	113 (57.9)	52 (61.9)	15 (62.5)	31 (60.8)	15 (41.7)	
Female	82 (42.1)	32 (38.1)	9 (37.5)	20 (39.2)	21 (58.3)	
Religion						0.327
Catholic	161 (82.6)	74 (88.1)	21 (87.5)	39 (76.5)	27 (75.0)	
Other religion	18 (9.2)	7 (8.3)	1 (4.2)	5 (9.8)	5 (13.9)	
None	16 (8.2)	3 (3.6)	2 (8.3)	7 (13.7)	4 (11.1)	
Family structure						0.012
Monoparental	45 (23.1)	28 (33.3)	3 (12.5)	10 (19.6)	4 (11.1)	
Extended	68 (34.9)	27 (32.1)	11 (45.8)	18 (35.3)	12 (33.3)	
Reconstituted	40 (20.5)	11 (13.1)	3 (12.5)	11 (21.6)	15 (41.7)	
Nuclear	42 (21.5)	18 (21.4)	7 (29.2)	12 (23.5)	5 (13.9)	
Schooling						0.979
Yes	135 (69.2)	59 (70.2)	17 (70.8)	35 (68.6)	24 (66.7)	
No	60 (30.8)	25 (29.8)	7 (29.2)	16 (31.4)	12 (33.3)	
Type of institution						0.019
Closed center	120 (61.5)	62 (73.8)	14 (58.3)	25 (49.0)	19 (52.8)	
Open center	75 (38.5)	22 (26.2)	10 (41.7)	26 (51.0)	17 (47.2)	
Duration in the institution	3.03 ± 2.12	3.41 ± 2.53	2.88 ± 1.56	2.33 ± 1.32	3.22 ± 2.15	Significant

Psychological characteristics and severity of sexual abuse

A significant association was observed between psychological distress and the severity of sexual abuse (χ² = 11.6; df = 3; p = 0.009). Post hoc analyses (adjusted residuals, α corrected = 0.00625) showed a significantly higher proportion of severe abuse among adolescents presenting with psychological distress (p = 0.0022).

Self-esteem was also associated with the severity of abuse (χ² = 24.54; df = 9; p = 0.004). Analyses of adjusted residuals (α corrected = 0.0031) showed that adolescents with very low self-esteem were significantly more exposed to severe abuse (p < 0.001).

No significant association was found between the attachment figure and the severity of sexual abuse (p = 0.095) (Table 2).

**Table 2:** Association between psychological characteristics and severity of sexual abuse

Variables	All (number (%))	Severity of sexual abuse					P
		None		Low	Moderate	Severe	
Psychological distress							0.009
Yes	125 (64.1)	48 (57.1)		12 (50.0)	34 (66.7)	31 (86.1)	
No	70 (35.9)	36 (42.9)		12 (50.0)	17 (33.3)	5 (13.9)	
Self-esteem							0.0035
Very low	48 (24.6)	16 (19.0)		3 (12.5)	10 (19.6)	19 (52.8)	
Low	70 (35.9)	29 (34.5)		8 (33.3)	24 (47.1)	9 (25.0)	
Medium	50 (25.6)	27 (32.1)		7 (29.2)	11 (21.6)	5 (13.9)	
High	27 (13.8)	12 (14.3)		6 (25.0)	6 (11.8)	3 (8.3)	
Attachment figure							0.095
Father	28 (14.4)	16 (19.0)		4 (16.7)	6 (11.8)	2 (5.6)	
Mother	81 (41.5)	31 (36.9)		10 (41.7)	21 (41.2)	19 (52.8)	
Third party	63 (32.3)	21 (25.0)		8 (33.3)	20 (39.2)	14 (38.9)	
Both parents	23 (11.8)	16 (19.0)		2 (8.3)	4 (7.8)	1 (2.8)	

Substance use and severity of sexual abuse

Consumption of alcohol, tobacco, and cannabis was reported by 127 (65.1%), 90 (46.2%), and 71 (36.4%) participants, respectively.

A significant association was observed between cannabis use and the severity of sexual abuse (χ² = 18.39; df = 3; p < 0.001). Post hoc analyses (adjusted residuals, α corrected = 0.00625) showed that approximately half of the adolescents who had experienced severe abuse consumed cannabis, a proportion significantly higher than in the other groups (p < 0.001).

Tobacco use was also associated with the severity of abuse (χ² = 9.85; df = 3; p = 0.019). However, adjusted residual analyses did not identify significant differences between categories after correction for multiple comparisons.

No significant association was observed between alcohol consumption and the severity of abuse (p = 0.314) (Table 3).

**Table 3:** Association between psychoactive substance use and severity of sexual abuse

Variables	All (number (%))	Severity of sexual abuse				P
		None	Low	Moderate	Severe	
Cannabis						0.000
Yes	71 (36.4)	17 (20.2)	9 (37.5)	27 (52.9)	18 (50.0)	
No	124 (63.6)	67 (79.8)	15 (62.5)	24 (47.1)	18 (50.0)	
Tobacco						0.020
Yes	90 (46.2)	30 (35.7)	9 (37.5)	30 (58.8)	21 (58.3)	
No	105 (53.8)	54 (64.3)	15 (62.5)	21 (41.2)	15 (41.7)	
Alcohol						0.314
Yes	127 (65.1)	59 (70.2)	12 (50.0)	32 (62.7)	24 (66.7)	
No	68 (34.9)	25 (29.8)	12 (50.0)	19 (37.3)	12 (33.3)	

Multivariate analysis

Ordinal logistic regression showed that the overall model was statistically significant (χ² = 59.24; df = 11; p < 0.001), indicating that the independent variables contribute to explaining the severity of sexual abuse.

Model fit tests were satisfactory (Pearson test: p = 0.740; deviance: p = 0.999), suggesting good model adequacy to the data. The Nagelkerke pseudo-coefficient of determination (R² = 0.284) indicated moderate explanatory power. The proportional odds assumption was met (χ² = 13.62; df = 22; p = 0.914), confirming the validity of the ordinal model.

After adjustment, several factors were significantly associated with a higher probability of belonging to a higher severity category of sexual abuse: female sex (aOR = 1.78; 95% CI: 1.00-3.16; p = 0.049); attachment to the mother (aOR = 5.12; 95% CI: 1.74-15.07; p = 0.003); attachment to a third person (aOR = 3.34; 95% CI: 1.06-10.49; p = 0.039); reconstituted (aOR = 5.19; 95% CI: 2.07-13.05; p < 0.001), extended (aOR = 2.35; 95% CI: 1.03-5.35; p = 0.041), and nuclear family structures (aOR = 2.57; 95% CI: 1.02-6.46; p = 0.045); and cannabis use (aOR = 3.49; 95% CI: 1.56-7.81; p = 0.002).

An inverse statistical association was observed between alcohol consumption and severity of sexual abuse in the adjusted model (aOR = 0.31; 95% CI: 0.16-0.63; p = 0.001). However, this finding must be interpreted with extreme caution and should not be interpreted as evidence of a protective effect.

Age and tobacco use were not significantly associated with the severity of sexual abuse after adjustment (p > 0.05) (Table 4).

**Table 4:** Factors associated with the severity of sexual abuse aOR: adjusted odds ratio, CI: confidence interval, Ref.: reference category

Variable	Modality	Β	SE	aOR	95% CI	p-value
Sex (Ref.: male)	Female	0.576	0.293	1.78	1.00-3.16	0.049
Age		0.119	0.096	1.13	0.93-1.36	0.216
Attachment (Ref.: both parents)	Mother	1.634	0.550	5.12	1.74-15.07	0.003
	Third person	1.205	0.585	3.34	1.06-10.49	0.039
Family structure (Ref.: single parent)	Nuclear	0.943	0.471	2.57	1.02-6.46	0.045
	Extended	0.856	0.419	2.35	1.03-5.35	0.041
	Reconstituted	1.646	0.470	5.19	2.07-13.05	<0.001
Alcohol (Ref.: no)	Yes	-1.159	0.353	0.31	0.16-0.63	0.001
Tobacco (Ref.: no)	Yes	0.572	0.368	1.77	0.86-3.64	0.120
Cannabis (Ref.: no)	Yes	1.249	0.412	3.49	1.56-7.81	0.002

Although attachment figures were not significantly associated with severity in bivariate analysis, the variable became significant after multivariate adjustment. This may reflect confounding or suppression effects related to the interrelationships between familial and behavioral variables included in the model. Therefore, this finding should be interpreted cautiously and requires confirmation in longitudinal studies.

## Discussion

This study aimed to identify factors associated with the severity of sexual abuse among vulnerable adolescents attending reception centers in Kinshasa. The results highlight a high prevalence of sexual abuse (56.9%), as well as significant associations between the severity of abuse and several sociodemographic, familial, psychological, and behavioral factors. However, due to the cross-sectional design of the study, these factors cannot be interpreted as causal relationships.

Prevalence of sexual abuse

The proportion of adolescents who experienced sexual abuse in this study is particularly high. This result is higher than some estimates from the international literature [4] but remains consistent with data reported in contexts of high social vulnerability, particularly among street children [20,21]. This high prevalence can be explained by cumulative exposure to high-risk environments, characterized by the absence of family protection, economic precariousness, and social disorganization [3,19,23].

Sociodemographic factors

Age was associated with the severity of abuse in bivariate analysis, but this association did not persist after adjustment. This result may be related to cumulative exposure to risk situations during adolescence, as already described in the literature [4,6].

Female sex appears as a factor associated with increased severity of abuse in multivariate analysis, which is consistent with international data showing greater vulnerability of girls to severe forms of sexual violence [4,7].

Furthermore, the type of center was associated with severity in bivariate analysis, with adolescents from open centers seeming more represented among severe forms. However, this association did not persist after adjustment, suggesting that it may be explained by confounding factors, particularly sociodemographic, familial, or behavioral factors. Thus, the type of center does not appear to be an independent factor of severity in our model. Nevertheless, it could indirectly reflect differentiated contexts of vulnerability, such as less supervision or increased exposure to environmental risks, as suggested in studies on street children [20].

Familial and attachment factors

Family structure appears as a variable strongly associated with the severity of sexual abuse. Adolescents from reconstituted or extended families present a higher probability of severe abuse, which aligns with studies showing that family instability and parental recompositions are associated with a higher likelihood of exposure [14-17].

The association between attachment to the mother or to a third person and the severity of abuse may seem counterintuitive. However, these results may reflect life trajectories marked by family breakdowns, placements, or situations of abandonment. Thus, these attachment figures could constitute indirect indicators of psychosocial vulnerability rather than protective factors [18,24].

Psychological factors

Psychological distress and low self-esteem are strongly associated with the severity of sexual abuse. These results are consistent with numerous studies showing that sexual abuse is associated with emotional disorders, particularly anxiety and depression, as well as impaired self-image [6,8].

Due to the cross-sectional nature of the study, the direction of the association cannot be established. Although psychological distress and low self-esteem were associated with severity in bivariate analysis, their temporal ambiguity and the possibility that they may reflect consequences rather than determinants did not allow their inclusion in the main multivariate model, limiting any causal interpretation [6,8].

Substance use

Cannabis use is significantly associated with increased severity of sexual abuse. This result is consistent with the literature, which identifies substance use as a behavior associated with a context of vulnerability, which may be related to impaired judgment and increased exposure to high-risk environments [9-13,22].

An inverse statistical association was observed between alcohol consumption and the severity of sexual abuse in the adjusted analysis. This finding is counterintuitive and should not be interpreted as evidence of a protective effect of alcohol consumption. Because of the cross-sectional design, the direction and temporality of the association cannot be established. In addition, residual confounding, reporting bias, and unmeasured contextual factors may explain this result. Therefore, this association should be considered exploratory and interpreted with extreme caution.

Strengths and limitations of the study

This study has several strengths, notably the use of internationally recognized and widely used assessment tools (CTQ-SF, GHQ-28, and RSES) and the use of an ordinal logistic regression model adapted to the nature of the dependent variable.

However, certain limitations must be highlighted. Due to the cross-sectional design, causal relationships cannot be established because exposure and outcome were measured simultaneously. Non-probability consecutive sampling conducted in only two reception centers belonging to the same organization (OSEPER) constitutes an important methodological limitation. Adolescents recruited from these centers may not be representative of all vulnerable adolescents or adolescents attending reception centers in Kinshasa or in the Democratic Republic of the Congo. In addition, selecting two centers managed by the same organization may have introduced institutional selection bias because participants may share similar environmental, educational, and psychosocial characteristics. Therefore, the findings should not be generalized beyond the specific context of this study.

A major limitation of this study is that the CTQ-SF, GHQ-28, and RSES have not undergone formal cross-cultural validation or psychometric adaptation in the Democratic Republic of the Congo. Although the questionnaires were administered in French and a preliminary pre-test confirmed item comprehension, no rigorous process of translation, back-translation, semantic equivalence assessment, cultural adaptation, or local psychometric validation was conducted. Consequently, measurement bias cannot be excluded, and all findings, including the observed associations, should be interpreted with substantial caution.

In addition, psychological variables such as psychological distress and self-esteem were excluded from the multivariate model because of temporal ambiguity and the risk of overadjustment. Although methodologically justified, this may have limited the exploration of clinically important associations.

Implications and perspectives

The results of this study underline the need to develop targeted interventions to prevent sexual abuse and reduce its severity among adolescents in street situations. Longitudinal studies are necessary to better understand causal relationships and the evolution of risk factors over time.

## Conclusions

This study highlights a high prevalence of sexual abuse among adolescents attending reception centers in Kinshasa. The severity of the abuse is independently associated with female sex, family context, attachment figures, and cannabis use. These results suggest that sexual abuse among vulnerable adolescents is linked to a complex interaction of social, family, and behavioral factors. They underscore the importance of implementing integrated interventions that combine prevention, early screening, psychosocial support, family-centered approaches, and the management of substance use.

From a clinical and public health perspective, these findings highlight the need to strengthen protection and care systems for adolescents in institutional settings. However, due to the cross-sectional nature of the study and the absence of formal cross-cultural validation of the assessment instruments in the Democratic Republic of the Congo, all findings should be interpreted with substantial caution. Despite these limitations, this work provides important data in a context that remains poorly documented in the Democratic Republic of the Congo and constitutes a useful foundation for guiding future research and interventions.
